# Aromatic aldehydes formed during heating of cinnamon-flavored e-liquids drive pro-inflammatory responses in human aortic smooth muscle cells

**DOI:** 10.1016/j.crtox.2026.100299

**Published:** 2026-05-10

**Authors:** Mariam Bitar, Jérémie Pourchez, Mohamad Sleiman, Laurent Bertoletti, Valérie Forest

**Affiliations:** a Mines Saint-Etienne, Univ Jean Monnet, INSERM, U 1059 Sainbiose, Centre CIS, F-42023 Saint-Etienne, France; b Université Clermont Auvergne, Clermont Auvergne INP, CNRS, ICCF, F-63000 Clermont-Ferrand, France; c Service de Médecine Vasculaire et Thérapeutique, CHU de Saint-Etienne, Saint-Etienne, France; d INSERM, UMR1059, Equipe Dysfonction Vasculaire et Hémostase, Université Jean-Monnet, F-42055 Saint-Etienne, France; e INSERM, CIC-1408, CHU Saint-Etienne F-42055 Saint-Etienne, France

**Keywords:** E-cigarettes, Cytotoxicity, Pro-inflammatory response, Cardiovascular effect, Flavor, Cinnamon, Aortic smooth muscle cells

## Abstract

Flavor additives can contribute to the biological effects of electronic cigarette (e-cigarette) aerosols, yet the mechanisms underlying flavor-related vascular toxicity remain poorly understood. We previously showed that exposure of human aortic smooth muscle cells (AoSMCs) to cinnamon-flavored e-cigarette aerosol induced a marked pro-inflammatory response that was amplified by heating. The present study aimed to identify thermal degradation products derived from cinnamon flavoring that may contribute to this effect. Aerosol condensates were generated from cinnamon-flavored e-liquids under controlled vaping conditions, and a chemical screening approach was used to identify degradation products. Benzaldehyde and phenylacetaldehyde were detected as specific to the heated cinnamon condensate and were subsequently selected for targeted *in vitro* testing. AoSMCs were exposed to aerosols, unheated e-liquids, or e-liquids supplemented with aldehydes (alone or in combination), and cytotoxicity and pro-inflammatory responses were assessed. Exposure to cinnamon aerosol induced a significant increase in IL-8 release without detectable cytotoxicity. Supplementation of e-liquids with benzaldehyde reproduced much of the IL-8 response observed with the aerosol, whereas phenylacetaldehyde alone elicited only a limited effect. Co-exposure to both aldehydes did not further enhance IL-8 production beyond that induced by benzaldehyde alone, suggesting potential interaction effects among cinnamon-derived degradation products.

Taken together, these findings indicate that thermal processing of cinnamon-flavored e-liquids generates aromatic aldehydes that contribute to pro-inflammatory signaling in human vascular smooth muscle cells, with benzaldehyde emerging as a major contributor under the present experimental conditions. This study highlights the importance of considering flavor-derived degradation products when evaluating the vascular toxicity of e-cigarette emissions.

## Highlights

• Flavor additives can impact the biological effects of electronic cigarette aerosols.
• We identified thermal degradation products derived from cinnamon flavored e-liquid.
• Benzaldehyde and phenylacetaldehyde were specific to the heated cinnamon condensate.
• Their *in vitro* toxicity was assessed on human aortic smooth muscle cells.
• Benzaldehyde was the primary contributor to the observed proinflammatory response.

## Graphical abstract

**Figure:**
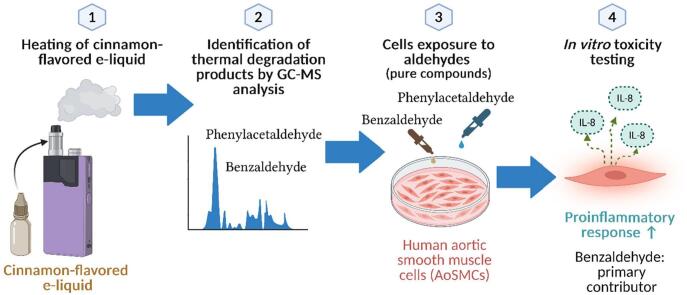

## 1 Introduction

The use of electronic cigarettes (or e-cigarettes) has increased significantly over the past decade, especially among young adults and adolescents. In France, e-cigarette use has increased notably in recent years: daily vaping among 17-year-olds rose from 1.9 % in 2017 to 6.2 % in 2022, and in 2023 current and daily use among 18–24-year-olds reached 11.1 % and 7.1 %, respectively (“Les drogues à 17 ans - Analyse de l’enquête ESCAPAD 2022,” 2023; “Tabagisme et vapotage parmi les 18–75 ans en 2023,” 2025). Marketed as a “less harmful” alternative to tobacco smoking, they have become popular due to the wide variety of flavors available, ranging from fruity to sweet and spicy blends (Notley et al., 2022).

Unlike tobacco products that rely on combustion, e-cigarettes generate an aerosol by heating a mixture of propylene glycol (PG), vegetable glycerin (VG), and optionally nicotine and flavorings. While this process avoids the formation of some toxic substances associated with tobacco combustion, the safety of the generated aerosols remains highly debated particularly regarding the possible chemical substances formed during e-liquid heating, whose toxicological effects are still only partially understood and assessed (Gordon et al., 2022). During aerosolization, the initial ingredients in e-liquids undergo chemical transformations, producing secondary compounds not present in the original formulation. Among these thermally-derived degradation products, volatile aldehydes (formaldehyde, acetaldehyde, and acrolein) are of particular concern due to their well-documented cellular and respiratory toxicity (Dupont and Aubin, 2019).

Several studies have shown that some flavorings can enhance the production of these aldehydes independently of dry-puff conditions, and that their emission increases significantly with device power (Khlystov and Samburova, 2016). For example, one study using gas chromatography-mass spectrometry (GC–MS) showed that flavorings such as menthol or linalool greatly increased the formation of reactive carbonyls and impaired the ability of cells to neutralize these species via glutathione (GSH) adduct formation (Chen et al., 2021). Similarly, a study testing four isolated flavoring compounds (cinnamaldehyde, eugenol, menthol, and vanillin) revealed the formation of several hundred degradation products, varying according to the flavor and heating temperature (250–750 °C) (Kuehl et al., 2022). In addition, it has been shown that chemical reactions such as acetal formation can occur directly in the e-liquid, even without heating, influenced by the PG/VG ratio, the presence of nicotine, and acidic compounds (Kerber and Peyton, 2022). One study also revealed that *trans*-cinnamaldehyde produces more toxic carbonyls in the presence of nicotine, unlike other flavorings whose toxicity is mitigated by nicotine (Kerber et al., 2022).

Beyond the formation of small volatile aldehydes from the PG/VG matrix, flavoring compounds may also generate specific degradation products upon heating. Cinnamaldehyde, a phenylpropene derivative commonly used as a cinnamon flavoring, is known to undergo thermal oxidation and structural rearrangements. Chemical studies have shown that its oxidation can lead to the formation of aromatic aldehydes, including benzaldehyde and phenylacetaldehyde, resulting from cleavage of the propenyl side chain at elevated temperatures. These compounds are not necessarily present in the original e-liquid formulation as ingredients, but may be formed as by-products during thermal processing, highlighting the contribution of cinnamon flavorings to the generation of distinct aldehydic species (Yu et al., 2020, Yu et al., 2021).

Among the most widely used flavorings in e-liquids, cinnamon (typically as *trans*-cinnamaldehyde) raises particular concern due to its high chemical reactivity and potentially harmful biological effects. Several studies have reported that this molecule is present at significant concentrations in commercial refill fluids and is efficiently transferred into the aerosol, making user exposure both real and potentially concerning (Behar et al., 2016). At the cellular level, cinnamaldehyde has been shown to profoundly disrupt innate respiratory function. It inhibits alveolar macrophage phagocytosis (Clapp et al., 2017), reduces phagocytic activity and oxidative burst in human neutrophils (Hickman et al., 2019), and decreases the ciliary beat frequency of human bronchial epithelial cells by impairing mitochondrial function and ATP production (Clapp et al., 2019). Additionally, cinnamaldehyde can spontaneously react with the PG/VG solvents in e-liquids to form acetals even without heating which may alter its toxicological profile (Kerber and Peyton, 2022). A recent study further demonstrated that cinnamaldehyde’s toxic effects on bronchial cells persist after heating, highlighting the importance of considering its thermal degradation products (Kerber et al., 2022).

Beyond its respiratory effects, recent findings indicate that cinnamaldehyde may also affect the cardiovascular system. Using human iPSC-derived cardiomyocytes, one study found that cinnamaldehyde, whether heated or unheated, impaired cellular contractility and calcium homeostasis, suggesting a potentially arrhythmogenic effect (Nystoriak et al., 2019). We have previously provided further insight into the cardiovascular effects of cinnamaldehyde using human aortic smooth muscle cells (AoSMCs). We first demonstrated that exposure to cinnamon-flavored e-cigarette aerosols, particularly those generated at high power, significantly increased secretion of the pro-inflammatory cytokine IL-8 compared with the corresponding unheated e-liquid, without inducing cytotoxicity or oxidative stress (Michon et al., 2022). These results suggest that certain compounds formed during the heating of cinnamaldehyde may trigger vascular inflammation. More recently we confirmed that cinnamon-containing condensates induced strong pro-inflammatory responses in AoSMCs, again measured via IL-8 release. At higher power settings, cytotoxic effects were also observed, highlighting the combined impact of flavor and heating temperature on vascular health (Bitar et al., 2025). These findings point to a targeted inflammatory response, possibly linked to specific cinnamon-related degradation products.

Despite these findings, few studies have systematically compared the chemical substances formed during heating and determined their exact origin whether they arise from the PG/VG matrix or are uniquely associated with the flavoring. This distinction is critical for identifying high-risk molecules, better understanding the toxicity mechanisms of e-cigarette use, and guiding safer e-liquid formulations. We hypothesized that specific aromatic aldehydes generated during thermal processing of cinnamon-flavored e-liquids are key contributors to the amplified pro-inflammatory response previously observed in AoSMCs. The present study aims to address these questions by combining analytical and toxicological approaches. Three types of samples were prepared: an unheated cinnamon-flavored e-liquid, a condensate generated from the same e-liquid under realistic heating conditions by a vaping product, and a condensate of PG/VG alone without any flavoring. Their chemical profiles were analyzed to identify heat-specific degradation compounds and determine their likely origin. In a second phase, selected compounds or extracts were tested on human aortic smooth muscle cells to assess their cytotoxic and pro-inflammatory potential. These results will help clarify the role of thermal degradation products in potential vascular toxicity linked to e-cigarette use, and inform future regulatory decisions regarding flavor additives.

## 2 Materials and methods

### 2.1 Experimental design

**Fig. 1:**
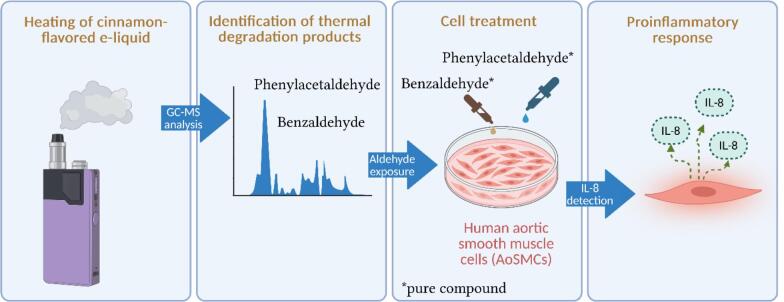
Schematic overview of the workflow used to identify cinnamon-derived thermal degradation products and assess pro-inflammatory responses in AoSMCs.

As shown by Fig. 1, the study was designed to identify chemical substances potentially formed as by-products during the thermal processing of a vaping device using a cinnamon-flavored e-liquid and to evaluate their contribution to vascular cell responses. To distinguish between compounds originating from the solvent matrix and those specifically associated with the cinnamon flavoring, three types of samples were prepared: 1) unheated cinnamon-flavored e-liquid, 2) aerosol condensate generated from a PG/VG base without flavoring, and 3) aerosol condensate generated from the same PG/VG base containing cinnamon flavoring (1.5 % (w/w) flavor in e-liquid). The PG/VG base (70/30) was purchased from Aromea (Saint-Aunès, France), and the cinnamon flavoring was supplied by Bio Concept (Niort, France).

The objective was to identify compounds specifically associated with heating the cinnamon flavoring by focusing on peaks present in the cinnamon condensate but absent from the unheated e-liquid and the PG/VG-only condensate. In our screening analysis, benzaldehyde and phenylacetaldehyde were identified as the relevant cinnamon-condensate-specific compounds and were therefore selected for subsequent *in vitro* testing. These compounds were tested on human aortic smooth muscle cells either individually or in combination, and compared with the biological effects of the parent e-liquid and the corresponding aerosol condensate. This comparative approach was used to determine whether a single compound, or a combination of compounds, was primarily responsible for the observed biological effects.

### 2.2 Aerosol generation and condensate collection

**Fig. 2:**
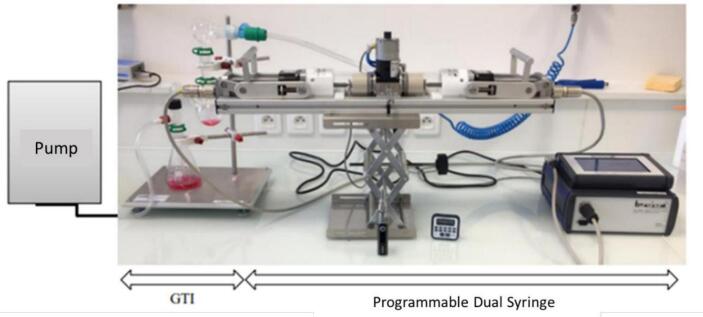
Setup used for e-cigarette aerosol generation using a programmable dual syringe pump and collection with a glass twin impinger (GTI).

Aerosols were produced using a GS Air 2 Atomizer (Eleaf, Shenzhen, China) equipped with a GS Air Series Atomizer Head (0.75 Ω) and powered by an iStick TC40W battery (Eleaf, Shenzhen, China). Aerosol generation was controlled and puffing reproducibility ensured, achieved with a Programmable Dual Syringe Pump (PDSP, Burghart Messtechnik GmbH, Wedel, Germany). The e-cigarette was positioned at a 30° angle to simulate the tilt during inhalation. Vaping parameters followed with the French Standardization Association (AFNOR) standard XP D90-300–3, corresponding to 3-second puffs of 55 mL each, taken every 30 s, with 20 puffs per series and two series separated by a 300-second interval, resulting in a total of 40 puffs per condition. The generated aerosols were collected using a Glass Twin Impinger (GTI, Copley Scientific Limited, Nottingham, UK) containing 30 mL of PBS in the lower chamber (Erlenmeyer flask), allowing collection of the respirable aerosol fraction. The GTI was connected to a low-capacity vacuum pump (Model LCP5, Copley Scientific Limited) operating at a flow rate of 60 L/min. The setup is presented in Fig. 2.

### 2.3 Gas chromatography–mass spectrometry (GC–MS) analysis

Samples were analyzed by GC–MS using a GC-2010 Plus/GQMS-QP2010 SE system (Shimadzu, Japan) equipped with a MEGA 5-MS capillary column (30 m × 0.25 mm, 0.25 μm film thickness). The oven temperature was programmed from 60 °C (held for 1 min) to 270 °C at a rate of 15 °C min⁻^1^, then held at 270 °C for 5 min. Injection was performed at 280 °C in split mode (5:1). The mass spectrometer source temperature was set at 230 °C and the transfer line temperature at 280 °C. Spectra were recorded over a mass-to-charge (*m*/*z*) range of 50–350. Identification of the major constituents was carried out by comparison with the NIST 17 database, considering compounds with a similarity score greater than 95 %, and confirmed when necessary using authentic standards. Semi-quantification was performed using cinnamaldehyde as the reference compound, and results were expressed as cinnamaldehyde equivalents.

### 2.4 Cell culture and exposure

Human aortic smooth muscle cells (AoSMCs) were purchased from Lonza (Basel, Switzerland) and maintained in smooth muscle cell growth medium-2 (SmGM, Lonza, Basel, Switzerland). This medium contains Fetal Bovine Serum (FBS), human fibroblast growth factors, epidermal growth factors, insulin, and gentamicin-amphotericin. Flasks were trypsinized when cells reached approximately 80 % confluence with trypsin (0.025 %)/EDTA (Lonza, Basel, Switzerland). Cells were then differentiated for 1–2 weeks in smooth muscle cell basal medium (SmBM, Lonza, Basel, Switzerland) supplemented with 5 % penicillin/streptomycin (VWR, Monroeville, PA, USA). Cultures were maintained in an incubator at 37 °C and in a 5 % CO_2_ atmosphere, and the culture medium was changed two or three times a week. Then, 96-well plates were coated with 3 μg/cm^2^ fibronectin (Sigma-Merck, Saint-Quentin-Fallavier, France), reconstituted with 1 mL phosphate-buffered saline (PBS, Fisher Scientific, Illkirch, France). Plates were air-dried for 45 min and stored for 2–4 weeks. 15,000 cells were seeded in 50 μL SmBM medium in each well of a 96-well plate which was incubated at 37 °C in 5 % CO_2_ for 24 h. The cells were then exposed to 150 μL of e-liquid or condensates for 24 h. The cells used for these experiments were at passage 7.

Cells were exposed to 128 µg of e-liquid or condensate per mL of culture medium. This concentration was chosen based on our previous work, which showed maximal pro-inflammatory responses at this level (Bitar et al., 2025). Two aldehydes, benzaldehyde and phenylacetaldehyde, identified as potential thermal degradation products of cinnamon flavoring, were selected for further testing and purchased as pure analytical standards from Sigma-Aldrich (Saint-Quentin-Fallavier, France). These compounds were spiked into the cinnamon-flavored e-liquid matrix either individually or in combination at four concentrations to establish a concentration–response relationship and enable standardized comparisons across conditions. For the combination experiments, the two compounds were mixed in equal parts; the indicated concentration refers to the final total aldehyde concentration. For example, a final aldehyde concentration of 0.5 ng/mL of culture medium corresponds to 0.25 ng of benzaldehyde per mL of culture medium plus 0.25 ng of phenylacetaldehyde per mL of culture medium.

To define a biologically relevant range of aldehyde concentrations, we first calculated the target aldehyde concentrations in the culture medium and used concentrations corresponding to approximately 0.3–26 times this value.

The target aldehyde concentration in the culture medium ([A]_medium_) was estimated using the following relationship:

$${\left[\text{A}\right]}_{\text{medium}}=\;{\left[\text{e}-\text{liq}\right]}_{\text{medium}}\times {\;\text{R}}_{\text{A}/\text{e}-\text{liq}}\times {\;\text{R}}_{\text{F}/\text{e}-\text{liq}}$$

where

• [e-liq]_medium_ is the concentration of e-liquid in the culture medium, which is defined as the mass of e-liquid per volume of culture medium. In our experiments this value was fixed at 128 µg/mL.
• R_A/e-liq_ is the aldehyde-to-e-liquid ratio, defined as the mass of aldehyde relative to the mass of flavor. This ratio was estimated from GC–MS semi-quantitative analysis (see Results section).
• R_F/e-liq_ is the flavor-to-e-liquid ratio, defined as the mass of flavor relative to the total e-liquid mass. The formulation used in this study contained 15 mg of flavor per g of e-liquid, so R_F/e-liq_ = 15/1000 = 1.5 %. In other words, the formulation contained 1.5 % (w/w) flavor in e-liquid.

### 2.5 Toxicological assays

#### 2.5.1 Cytotoxicity − LDH test

To assess cell membrane damage from exposure to e-liquids or aerosol condensates, the release of lactate dehydrogenase (LDH) into the culture supernatant was measured after a 24-h incubation period using the CytoTox-96 assay (Promega, Madison, WI, USA) according to the manufacturer's instructions (Cervellati et al., 2014, Effah et al., 2023, Michon et al., 2022). The optical density of the samples was then determined using a microplate reader set to 490  nm (Multiskan GO, ThermoFisher Scientific, Waltham, MA, USA) and compared with that of the control group (unexposed cells). A positive control representing the maximum LDH release (100 %) after cell lysis was used.

#### 2.5.2 Pro-inflammatory response − Interleukin-8 test

After exposure, 50 µL of cell culture supernatant from each well was used for Interleukin-8 (IL-8) quantification using the human IL-8 ELISA Kit (ThermoFisher Scientific, Waltham, MA, USA) following the manufacturer's guidelines. Absorbance was measured at 450  nm with a microplate reader (Multiskan GO, ThermoFisher Scientific, Waltham, MA, USA) (Sinha et al., 2022, Staal et al., 2023, Vasanthi Bathrinarayanan et al., 2018). IL-8 levels were then compared to those of the control group, consisting of unexposed cells.

### 2.6 Statistical analysis

Analyses and graphs were performed on Prism software (GraphPad, San Diego, CA). Statistical significance was declared when *p* < 0.05, as calculated with two-way ANOVA tests and Tukey post-tests. Each data point represents the mean of three independent experiments each performed in quaduplicate and is presented with the arithmetic standard error of the mean (± SEM).

## 3 Results

### 3.1 Chemical analysis and by-products identification

**Fig. 3:**
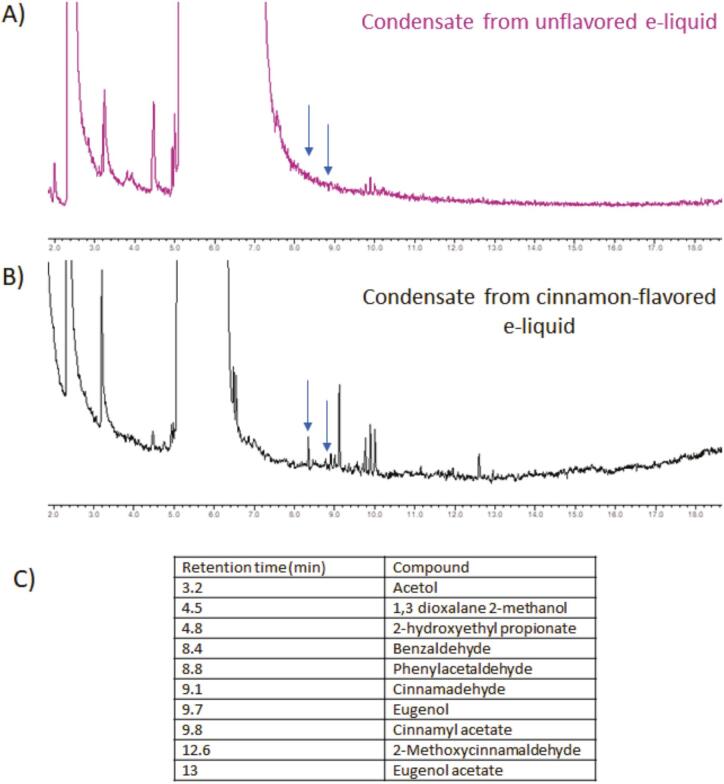
Chromatograms of condensates generated from an unflavored e-liquid (A) and a cinnamon-flavored e-liquid (B). C) Retention times and compounds identified in the condensate from a cinnamon-flavored e-liquid. Arrows at 8.4 and 8.8 min on the chromatograms indicate where the peaks for benzaldehyde and phenylacetaldehyde, respectively, are expected.

Chromatographic analysis of the three samples showed that benzaldehyde and phenylacetaldehyde were detectable as trace compounds in the cinnamon-containing condensate (Fig. 3), but were not detected in the corresponding unheated e-liquid used as a control to identify potential by-products of the cinnamon flavoring. These two compounds were therefore considered representative cinnamon-derived thermal degradation products and selected for subsequent *in vitro* toxicology testing. In addition, the chemical analysis confirmed the presence of several major flavor-related constituents in the cinnamon-flavored formulation including cinnamaldehyde (the main cinnamon flavoring ingredient) and related aromatic compounds, consistent with the chromatographic profiles provided.

Regarding relative abundance, for most detected constituents, concentrations in the condensate were typically lower than in the unheated e-liquid by a factor of approximately 5–10, with an average factor of about 8. Based on these chemical analysis data, approximate working concentrations were estimated for selected by-products to guide the design of exposure conditions. A semi-quantitative analysis using the areas under the curves determined that benzaldehyde and phenylacetaldehyde were present at similar orders of magnitude, with an estimated aldehyde-to-e-liquid ratio R_A/e-liq_ ranging from 0.05 % to 1 % (w/w). We then calculated the concentration of aldehyde in the culture medium as previously defined: [A]_medium_ = [e-liq]_medium_ x R_A/e-liq_ x R_F/e-liq_. Therefore, [A]_medium_ ranged from 0.96 to 1.9 ng of aldehyde per mL of culture medium (128 x 0.05 % x 1.5 % = 0.96 and 128 x 1 % x 1.5 % = 1.9). Based on this estimated range, four concentrations of aldehydes were selected for *in vitro* testing: 0.5, 2.5, 7.5 and 25 ng/mL. These concentrations were added to the culture medium together with 128 µg/mL of e-liquid, and tested either individually or in combination.

In summary, human aortic smooth muscle cells were exposed to five experimental conditions: (1) PG/VG (70/30) e-liquid containing cinnamon flavoring; (2) condensate generated from PG/VG (70/30) e-liquid containing cinnamon flavoring; (3) PG/VG (70/30) e-liquid supplemented with benzaldehyde; (4) PG/VG (70/30) e-liquid supplemented with phenylacetaldehyde; and (5) PG/VG (70/30) e-liquid supplemented with a combination of benzaldehyde and phenylacetaldehyde. These exposure conditions were designed to compare the effects of the cinnamon-flavored e-liquid before and after heating, and to evaluate the contribution of selected cinnamon-derived degradation products to the observed cellular responses.

### 3.2 Cytotoxicity and pro-inflammatory response

**Fig. 4:**
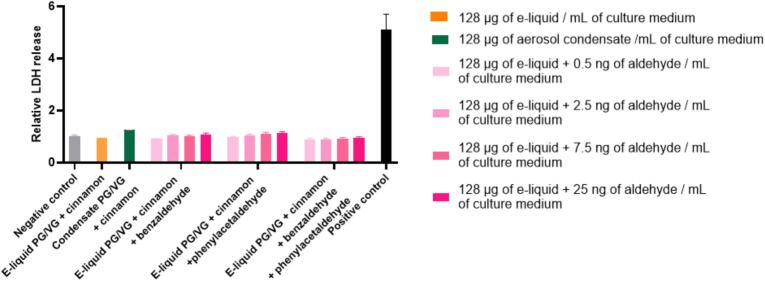
Relative cytotoxicity induced by the different experimental conditions assessed after 24 h of cell exposure through LDH release. Results are expressed relative to control (unexposed) cells and are means of three independent experiments, each performed in quadruplicate. Statistical significance was determined with a two-ways ANOVA followed by Tukey’s post-hoc test between control and experimental groups. For inter-group comparisons, a two-ways ANOVA followed by Tukey’s post-hoc was also performed. No statistically significant increase in LDH release was observed in treated groups under the tested conditions.

We investigated the effects of cinnamon thermal degradation products on the cytotoxicity and pro-inflammatory responses of human aortic smooth muscle cells. Fig. 4 shows LDH release measured after 24 h of exposure to cinnamon-flavored e-liquids (PG/VG 70/30), their corresponding aerosol condensates, and e-liquids enriched with benzaldehyde, phenylacetaldehyde, or both. LDH release was not significantly increased compared with unexposed control cells, regardless of the condition or concentration tested. Exposure to cinnamon-flavored e-liquid, the corresponding condensate, or e-liquids enriched with benzaldehyde, phenylacetaldehyde, or a combination of both did not induce cytotoxicity, with LDH levels remaining close to baseline.

**Fig. 5:**
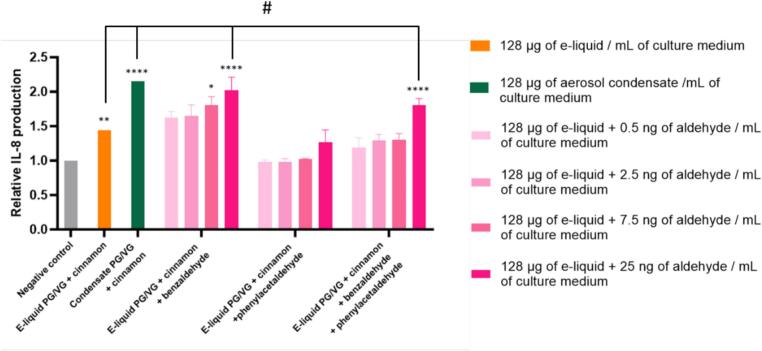
Relative pro-inflammatory response induced by the different experimental conditions assessed after 24 h of cell exposure through IL-8 production. Results are expressed relative to control (unexposed) cells and are means of three independent experiments, each performed in quadruplicate. Statistical significance is also indicated: *p < 0.05, **p < 0.01, ***p < 0.001, ****p < 0.0001 as determined with a two-ways ANOVA followed by Tukey’s post-hoc test between control and experimental groups. For inter-group comparisons, a two-ways ANOVA followed by Tukey’s post-hoc was also performed and indicated by: #p < 0.05.

Fig. 5 shows IL-8 production under the same exposure conditions. We found that exposure to unheated cinnamon-flavored e-liquid induced a moderate but significant increase in IL-8 production compared to unexposed control cells. In contrast, exposure to the condensate generated by the cinnamon-flavored e-liquid resulted in a significantly higher IL-8 release, considerably greater than in the control conditions and with the unheated e-liquid. Adding benzaldehyde to cinnamon-flavored e-liquid induced a dose-dependent increase in IL-8 production, with significant effects at the highest concentrations. In comparison, adding phenylacetaldehyde alone induced only a small increase in IL-8 levels at the tested concentrations. Notably, co-exposure to benzaldehyde and phenylacetaldehyde resulted in a marked increase in IL-8 production, particularly at the highest tested concentration, reaching levels comparable to those observed after exposure to cinnamon condensate. Statistical analysis revealed significant differences between unheated cinnamon-flavored e-liquid and cinnamon condensate, as well as between unheated cinnamon-flavored e-liquid and unheated cinnamon-flavored e-liquid with added benzaldehyde or the combination benzaldehyde and phenylacetaldehyde. Overall, these results suggest that benzaldehyde appears to be a major contributor of the pro-inflammatory activity observed with the cinnamon condensate, whereas phenylacetaldehyde alone appears to play a more limited role under our experimental conditions.

## 4 Discussion

In this *in vitro* study, we evaluated the cytotoxicity and pro-inflammatory response induced by thermal degradation products from cinnamon flavoring on human aortic smooth muscle cells. Previous work from our laboratory showed that exposure to a cinnamon-flavored e-liquid induces a moderate pro-inflammatory response, which is significantly amplified after heating, as indicated by the increased IL-8 production following exposure to the corresponding aerosol condensate (Bitar et al., 2025, Michon et al., 2022). The objective and originality of this study were to identify the thermal degradation by-products derived from cinnamon, that may be responsible for amplifying the inflammatory response after heating. By combining chemical analysis with targeted *in vitro* toxicological tests, we also demonstrate that certain aromatic aldehydes formed during heating, particularly benzaldehyde, contribute to this pro-inflammatory effect. Although IL-8 is not a direct marker of vascular dysfunction, its sustained induction in AoSMCs is relevant given its role in leukocyte recruitment and vascular remodeling.

Importantly, the biological activity of cinnamon-related e-cigarette constituents has also been reported in other *in vitro* models, indicating that the pro-inflammatory potential observed in AoSMCs is not limited to vascular cells. Using condensed aerosols generated from flavored e-liquids, Bengalli et al. showed that flavor composition strongly modulates toxicity in lung-relevant systems, with cinnamon-containing condensed aerosols among those producing the most pronounced adverse effects on epithelial viability and respiratory barrier integrity, along with detectable changes in inflammatory endpoints (including IL-8) (Bengalli et al., 2017). In immune cells, Muthumalage et al. reported that exposure of human monocytic cell lines to commonly used flavoring chemicals including cinnamaldehyde and to flavored e-liquids (without nicotine) induced dose-dependent IL-8 secretion and oxidative responses, indicating that cinnamon-related chemicals can directly promote inflammatory signaling in non-vascular cell types (Muthumalage et al., 2018). Finally, in a large-scale, open-source high-throughput screening approach, Sassano et al. found that e-liquids are highly heterogeneous in toxicity and that the presence (and concentration) of cinnamaldehyde were associated with higher toxicity values, reinforcing the view that cinnamon-related constituents are among the flavoring agents most consistently linked to adverse cellular responses (Sassano et al., 2018).

Among the compounds detected exclusively in the cinnamon condensate, benzaldehyde and phenylacetaldehyde were identified as the main cinnamon-derived thermal degradation products, consistent with literature data. Chemical studies have shown that cinnamaldehyde is prone to oxidation and thermal transformation, yielding aromatic aldehydes such as benzaldehyde and phenylacetaldehyde as major oxidation products (Yu et al., 2020). Additionally, research on the oxidation pathways of 3-phenyl-2-propene compounds (including cinnamaldehyde) supports the formation of benzaldehyde through oxidative cleavage reactions under elevated temperature or oxidative conditions, with phenylacetaldehyde also reported as an intermediate or product within these pathways (Yu et al., 2021).

When tested individually, benzaldehyde induced a concentration-dependent increase in IL-8 production in AoSMCs. This finding aligns with the broader literature which indicates that benzaldehyde is a relevant and potentially bioactive constituent of flavored e-cigarette emissions. Notably, benzaldehyde has been reported at high levels in e-cigarette aerosols, with the highest emissions measured in cherry-flavored products, highlighting the potential for substantial inhalation exposure (Kosmider et al., 2016). Supporting its direct pro-inflammatory potential, aerosol exposure of murine macrophages at the air–liquid interface to PG/VG containing benzaldehyde significantly increased keratinocyte-derived chemokine (KC; CXCL1) release compared with air and PG/VG alone, indicating inflammatory signaling in response to benzaldehyde delivered as an aerosol (Lamb and Rahman, 2023). Furthermore, nose-only inhalation exposure to cherry-flavored e-cigarettes has been shown to induce lung inflammation in mice, with increases in inflammatory markers and chemokines reported in lung tissue, supporting the biological plausibility of inflammatory outcomes following inhalation of benzaldehyde-associated flavor aerosols (Lamb et al., 2022).

In contrast, phenylacetaldehyde may be less potent under the conditions tested. Available data on phenylacetaldehyde are limited, but suggest model- and stimulus-dependent effects on inflammatory pathways rather than a uniform pro-inflammatory profile. For example, in keratinocytes and monocytes stimulated with *Cutibacterium acnes*, phenylacetaldehyde was reported to attenuate inflammatory responses, including reductions in pro-inflammatory cytokine output (Lee et al., 2025). In addition, phenylacetaldehyde has been shown to induce reactive oxygen species (ROS) and to modulate the Stat3/IL-6 axis in other cellular models (Choi et al., 2020), indicating biological activity that may not necessarily result in a strong IL-8 response in AoSMCs under the present exposure conditions.

Importantly, because vaping generates complex mixtures of co-occurring degradation products, evaluating single compounds may underestimate or misrepresent the biological impact of combined exposures. In our study, benzaldehyde largely recapitulated the IL-8 response observed with the cinnamon condensate, whereas phenylacetaldehyde alone produced only a limited effect. Notably, co-exposure to both aldehydes did not increase IL-8 production beyond the response induced by benzaldehyde alone and, in our conditions, appeared to attenuate it, suggesting a potential inhibitory interaction between cinnamon-derived degradation products. Together, these findings highlight the need to assess mixtures, as interactions between co-emitted compounds may modulate inflammatory responses in ways that cannot be predicted from single-compound testing (Marescotti et al., 2020, Strongin et al., 2025). It would be interesting to use mathematical models (such as Bliss independence or Loewe additivity) to better characterize the type of interaction occurring between compounds.

A key observation of this study is that increased IL-8 production occurred without a corresponding increase in LDH release. In all tested conditions, including the cinnamon condensate and aldehyde-supplemented e-liquids, LDH levels remained close to baseline. This dissociation between pro-inflammatory signaling and cytotoxicity confirms our previous observations in human aortic smooth muscle cells, where cinnamon-flavored aerosol condensates elicited a robust IL-8 response without substantial LDH release (Bitar et al., 2025, Michon et al., 2022). Taken together, these findings indicate that, under the present *in vitro* conditions, cinnamon-related emissions can trigger a pro-inflammatory response in AoSMCs without detectable cytotoxicity, as assessed by LDH.

This study has several limitations that should be acknowledged. First, the chemical characterization of cinnamon-related degradation products was based on a screening chemical approach primarily intended for qualitative comparison and compound selection, rather than comprehensive quantitative analysis. Therefore, the full spectrum of carbonyls reported in the literature may not have been captured. In particular, highly volatile low-molecular-weight aldehydes (e.g., formaldehyde and acetaldehyde) typically require carbonyl-specific derivatization using 2,4-dinitrophenylhydrazine (DNPH) followed by HPLC-based analysis for reliable detection and quantification, and certain thermally labile species may degrade during GC injection. Consequently, while benzaldehyde and phenylacetaldehyde were identified as trace compounds specific to the cinnamon condensate and were prioritized for biological testing, future studies would benefit from dedicated targeted analytical workflows to provide a more exhaustive and quantitative profile of carbonyl formation during aerosolization. Moreover, because only two candidate degradation products were prioritized for biological testing, the contribution of other cinnamon-related transformation products and mixture interactions cannot be excluded.

Second, the *in vitro* exposure model used here relies on a single vascular cell type and simplified exposure conditions. AoSMCs are a relevant target for assessing vascular inflammatory signaling, but they do not fully reproduce the multicellular complexity of the vascular wall or the dynamic interactions between endothelial cells, smooth muscle cells, and immune cells that occur *in vivo*. Moreover, the use of aqueous exposures to e-liquids or condensates cannot fully recapitulate aerosol deposition patterns, dose rates, or metabolism following inhalation. Incorporating complementary models such as endothelial cells, co-cultures, or more physiologically relevant exposure systems, would strengthen translational relevance. In addition, although the e-liquid/condensate matrix concentration was standardized, aldehyde spiking levels were selected to reflect the expected order of magnitude and to support a practical concentration–response assessment. Future studies using targeted quantitative carbonyl methods would help refine exposure estimates and strengthen exposure–response relationships. Finally, the inflammatory response was assessed using IL-8 as a single marker of pro-inflammatory activation. Although IL-8 is relevant in AoSMCs, it does not fully capture the broader inflammatory response. Future studies should therefore investigate additional cytokines, chemokines, and signaling pathways to provide a more comprehensive evaluation of cinnamon-related vascular inflammation. Similarly, further analyses would benefit from including earlier time points (*e.g.*, 2–6 h), which could provide additional insight into the kinetics of IL-8 induction.

Despite these limitations, this study provides evidence that thermal processing of cinnamon-flavored e-liquids is associated with an enhanced pro-inflammatory response in AoSMCs and supports a contributory role for selected aromatic aldehydes formed during heating, particularly benzaldehyde. Along with previous reports on cinnamon aerosol condensates, our findings highlight the importance of considering flavor-derived degradation products and mixture effects when evaluating the vascular toxicity of e-cigarette emissions. Improved quantitative chemical characterization, together with more physiologically relevant exposure systems such as air–liquid interface models enabling direct aerosol exposure, will be essential to better define exposure–response relationships and inform regulatory decisions regarding flavor additives.

## 5 Conclusion

In conclusion, these findings confirm our previous observations that heating cinnamon-flavored e-liquids is associated with an enhanced pro-inflammatory response in human aortic smooth muscle cells, as indicated by increased IL-8 release, while no detectable cytotoxicity was observed by LDH assay. Chemical screening identified benzaldehyde and phenylacetaldehyde as trace compounds specific to the cinnamon condensate, and targeted testing indicated that benzaldehyde appears to be a major contributor to IL-8 induction under our experimental conditions. These findings support the relevance of flavor-derived thermal degradation products in shaping the vascular toxicity of e-cigarette aerosols. Further studies using quantitative carbonyl analysis are warranted to refine exposure response relationships and support risk assessment of flavor additives.

**Funding**

This research did not receive any specific grant from funding agencies in the public, commercial, or not-for-profit sectors.

## CRediT authorship contribution statement

**Mariam Bitar:** Conceptualization, Formal analysis, Investigation, Visualization, Writing – original draft. **Jérémie Pourchez:** Conceptualization, Supervision, Writing – review & editing. **Mohamad Sleiman:** Formal analysis, Investigation, Writing – review & editing. **Laurent Bertoletti:** Supervision, Writing – review & editing. **Valérie Forest:** Conceptualization, Supervision, Writing – review & editing.

## Declaration of competing interest

The authors declare that they have no known competing financial interests or personal relationships that could have appeared to influence the work reported in this paper.
